# Dataset of cow manure by earthworm bio-composting process

**DOI:** 10.1016/j.dib.2021.106966

**Published:** 2021-03-18

**Authors:** Shuai Luo, Ruizhi Wei, Zicheng Qi, Qichao Zhang, Kaifen Wang

**Affiliations:** a Shandong Academy of Agricultural Machinery Sciences, Jinan 250100, China; b Donge County Inspection and Testing Center, Liaocheng 252200, China; c Agricultural Comprehensive Service Center of Fangcheng, Linyi 273406, China

**Keywords:** Vermicompost, Compost, Data collection system, Field experiments

## Abstract

Earthworm bio-composting is an environmentally friendly way of processing agricultural organic waste, especially cow manure. In order to observe the temperature, humidity and conductivity of cow manure during the biological composting process of earthworms, a composting data collection system was designed to collect the above data. The experiment was carried out in an earthworm breeding farm in Changqing District, Jinan City, and lasted for 50 days, from October 21, 2020 to December 10, 2020. The experiment data can be used for data comparison with conventional composting, and can also provide a reference for the exploration of the earthworm composting process.

## Specifications Table

**Table:** 

Subject	Agriculture Engineering
Specific subject area	Monitoring data of the process of using earthworm bio-composting of cow manure
Type of data	Table
How data were acquired	Environment temperature, stack temperature, humidity and conductivity was measured by sensors. 20 input signals can be collected by the paperless recorder at the same time. One or two channels are occupied by the sensor, depending on the amount of data collected. The current in the range of 4–20 mA was output by the sensor. The name and parameters of the signal were changed and input by the paperless recorder, according to the corresponding sensor performance parameters. The data saving interval was set to 1 min, which means that the data collected by the sensor would be saved in the internal memory of the paperless recorder every 1 min. The collected data exported by the paperless recorder was transferred to a separate USB flash drive after the experiment was completed.
Data format	Raw Analyzed
Parameters for data collection	The experiment site was randomly selected in the breeding beds, and each data setting was repeated. The experiment was started after the new cow manure was added. The data was collected by the sensor, and the paperless recorder automatically recorded at an interval of 1 min.
Description of data collection	The experiment time covered the complete cycle of earthworm reproduction. The data acquisition sensor was calibrated by the manufacturer before leaving the factory. A set of composting data acquisition system was designed through programming. Various sensors were connected with the paperless recorder. The electrical signal data obtained by the sensors was collected and transformed by the paperless recorder and stored in the random access memory.
Data source location	Institution: Jinan Hongyuan Earthworm Farming Cooperative City/Town/Region: Jinan Country: China Latitude and longitude (and GPS coordinates, if possible) for collected samples/data: 36°33′N, 116°41′E
Data accessibility	Repository name: Mendeley Data Data identification number: http://dx.doi.org/10.17632/4fg3htnbs9 Direct URL to data: http://dx.doi.org/10.17632/4fg3htnbs9

## Value of the Data

• This dataset provides data of dynamics in temperature, moisture content and electrical conductivity in the process of cow manure using earthworm bio-compost during a composting cycle, which is widespread but lacks attention.
• The data is useful for researchers who are concerned about the changes in the biological composting process, and can be used as a reference for studying how to improve the efficiency of bio-composting of cow manure with earthworms.
• This dataset can be used to assist in judging when to add water, harvest the earthworms, and take heat preservation measures during the production process of bio-composting from cow manure using earthworms..

## 1 Data Description

This article includes the raw data and analyzed data. The dataset provides data of dynamics in temperature, moisture content and electrical conductivity in the process of cow manure using earthworm bio-compost. The experiment lasted from October 21, 2020 to December 10, 2020, and there were complete records of data throughout the day from October 22 to December 9. As the temperature was too low after December, it was no longer suitable for the survival of earthworms in the field [1]. The raw data obtained through the self-made compost data collection system are available on Mendeley dataset.

**Fig. 1:**
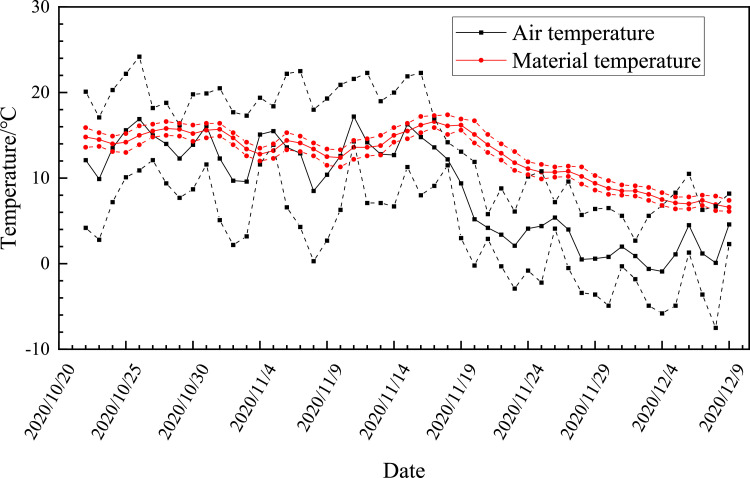
Changes in air temperature and material temperature during the composting process.

Fig. 1 shows the daily changes in air temperature 50–100 cm above the accumulation and 10–20 cm inside the accumulation. The arithmetic average of the complete data of each day were taken as the average of the temperature of the day. The red data point is the temperature inside the accumulation body, and the black data point is the air temperature above the accumulation body. The daily temperature is obtained by taking the arithmetic average of all the temperatures collected on the day. The lines of the same color above and below each solid line are the maximum and minimum temperature of the day.

**Fig. 2:**
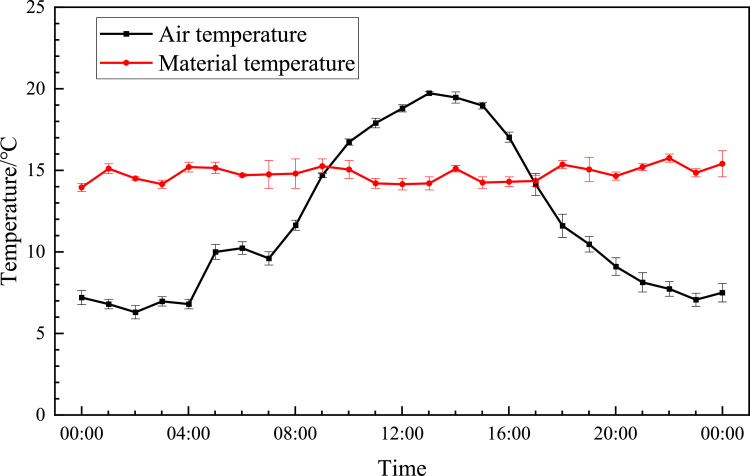
Changes in air temperature and material temperature in a day.

Fig. 2 shows the changes in air temperature 50–100 cm above the accumulation and 10–20 cm inside the accumulation on a random day (2020.11.14). The red data point is also the temperature inside the stack, and the black data point is the air temperature above the stack. The statistical time interval is 1 h. The temperature was highest at around 1:00pm, and the lowest at around 2:00am, with a temperature difference of 13.3 °C, and the temperature change basically followed the normal distribution trend. However, the temperature inside the material was kept very stable, and fluctuates little with temperature changes, and the maximum temperature difference was only 1.7 °C.

**Fig. 3:**
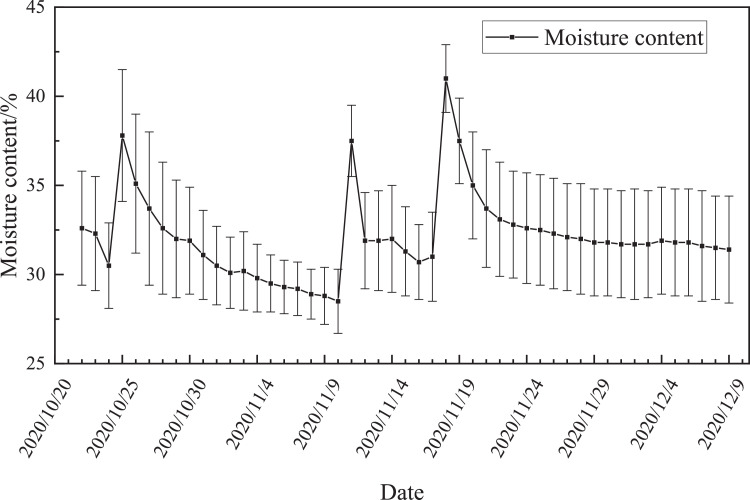
Change trend of material water content.

Fig. 3 shows the trend of the moisture content of the compost material over time. The three sharp increases in the moisture content were due to watering operations that day. After each watering, the moisture content of the material slowly droped to about 30% depending on the weather and could be maintained for a long time.

**Fig. 4:**
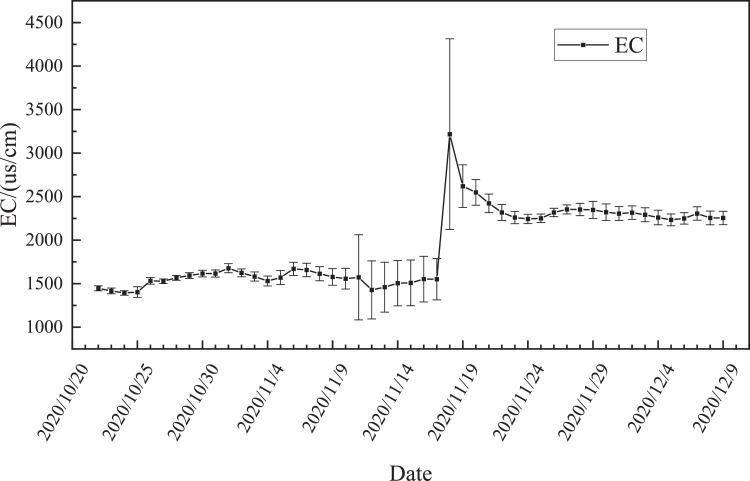
EC of the material changes over time.

Fig. 4 shows the trend of the conductivity of the compost material over time. The electrical conductivity of the material changed basically smoothly. Among them, there was a large fluctuation after watering on November 11, and it increased again after watering on November 18, and then slowly decreased and remained stable [2].

## 2 Experimental Design, Materials and Methods

### 2.1 Site description

The use of earthworms for bio-composting of cow manure, or the use of cow manure for earthworm breeding, in northern China, many of them are carried out under the woods. The experiment was conducted in the Hongyuan Earthworm Farming Cooperative in Changqing District, Jinan City, Shandong Province, China (36°33′N, 116°41′E). The earthworm breeding beds were strip-shaped along the planting direction of the trees. The upper surface of the cross-section of the breeding beds was arc-shaped, about 30 cm high and 80cm-100 cm wide. The breeding beds were equipped with spray pipes to maintain the proper humidity of the materials. The shelter of the woods can prevent the temperature of the breeding beds from being too high or too low. The consumption of cow manure was about 320 m^3^/hm, and the amount of earthworms used per hectare was about 300 kg.

### 2.2 Experimental design

The experiment was conducted from October 21, 2020 to December 10, 2020. After the adult earthworms of the previous season were harvested by a special cylindrical drum sieve with a flared opening, the remaining juvenile earthworms were evenly scattered on the breeding beds covered with cow manure. These earthworms would soon get into cow manure and started to grow and reproduce, digest the cow manure and excrete the vermicompost. A self-designed data acquisition system for data acquisition was used in the experiment. The data acquisition system was mainly composed of a paperless recorder and related sensors. AC 220 V voltage was used to power the equipment, which was equipped with a leakage protection switch, a surge protector and an IP54 protection level distribution box to ensure safety. DC 24 V voltage was used to power the sensor. A compost data acquisition system was designed for data acquisition. Multiple air temperature sensors, soil temperature and humidity sensors and Soil EC sensors was contained in this system. The paperless recorder and sensors parameters used are shown in Table 1.

**Table 1:** Main structure diagram of data acquisition system.

Serial				
number	Name	Manufacturer	Model	amount
1	Paperless recorder	Ningbo Keshun Instrument Co.,Ltd	KSR20A0R	1
2	Temperature sensor	Hangzhou Asmik Sensors Thchnology Co.,Ltd	MIK-WZP Pt100	3
3	Soil temperature and humidity sensor	Shandong Vemsee Technology Co., Ltd	VMS-3000-TR-*	2
4	Soil EC sensor	Shandong Vemsee Technology Co., Ltd	VMS-3000-ECH-*	2

### 2.3 Materials and methods

Earthworm bio-composting is an environmentally friendly way of processing agricultural organic waste, especially cow manure [3], [4]. The cow manure raw materials used in the experiment came from a nearby cattle farm and was left naturally for 2 weeks. The earthworm species used was *Eisenia fetida* [5], [6], which is widely used worldwide.

After the previous earthworm harvest, the cloth machine was used to evenly spread the cow manure on the earthworm breeding beds and then the experiment was started. Sensors for measuring temperature, humidity, and EC of composting materials were embedded in the materials to a depth of about 10–20 cm and fully contacted with the materials. The sensors that measures the air temperature were placed in the air at a height of 50–100 cm from the surface of the compost materials. The relevant parameters of the paperless recorder according to the sensor parameters were set to suit the measurement requirements. The automatic data saving interval was set to 1 min, that is, obtain analog data from the sensor every minute and save it in the built-in flash memory of the paperless recorder.

## Ethics Statement

None.

## CRediT Author Statement

**Shuai Luo:** Conceptualization, Methodology, Software, Validation, Formal analysis, Investigation, Data Curation, Writing - Original Draft, Writing - Review & Editing, Supervision, Funding acquisition; **Ruizhi Wei:** Formal analysis, Investigation, Resources, Data Curation, Writing - Review & Editing, Visualization; **Zicheng Qi:** Investigation, Resources, Funding acquisition; **Qichao Zhang:** Investigation, Data Curation, Writing - Review & Editing; **Kaifen Wang:** Data Curation, Writing - Review & Editing.

## Declaration of Competing Interest

The authors declare that they have no known competing financial interests or personal relationships which have or could be perceived to have influenced the work reported in this article.

## References

[1] Uvarov A.V., Tiunov A.V., Scheu S. (2011). Effects of seasonal and diurnal temperature fluctuations on population dynamics of two epigeic earthworm species in forest soil. Soil Biol. Biochem..

[2] Belmeskine H., Ouameur W.A., Dilmi N., Aouabed A. (2020). The vermicomposting for agricultural valorization of sludge from Algerian wastewater treatment plant: impact on growth of snap bean Phaseolus vulgaris L. Heliyon.

[3] Yadav K.D., Tare V., Ahammed M.M. (2010). Vermicomposting of source-separated human faeces for nutrient recycling. Waste Manag..

[4] Chaoui H., Keener H.M. (2008). Separating earthworms from organic media using an electric field. Biosyst. Eng..

[5] Liu F., Zhu P., Xue J. (2012). Comparative study on physical and chemical characteristics of sludge vermicomposted by Eisenia Fetida. Procedia Environ. Sci..

[6] Bhat S.A., Singh J., Vig A.P. (2016). Effect on growth of earthworm and chemical parameters during vermicomposting of pressmud sludge mixed with cattle dung mixture. Procedia Environ. Sci..

